# The Registration of Nursing Homes

**Published:** 1907-12-07

**Authors:** 


### Nursing Administration.

### THE REGISTRATION OF NURSING HOMES.

### IV. A QUESTION OF RESPONSIBILITY.

It is almost incredible that the superintendence of ursing homes should be open to any person endowed ith a capital of from £50 to £5,000, irrespective together of training or qualifications. Yet such is e case. One has only to look down the advertising lumns of nursing and medical papers to see one ursing home after another offered to the highest dder. They are always in the market, and those ho desire to dispose of them have small inclination examine into the fitness of applicants to carry on e business they wish to dispose of. Inquiries are nfined solely to their fitness to carry out the ancial obligations imposed by the terms of the reement, and should the intending purchaser show idence of being able to pay the price, who has the ght to demand more? No licence is required ch as would be demanded if, for instance, a pawn-op or public house were to change hands. The affic in the lives of men which goes on in these tablishments is considered to be beneath the attention of the law. Is it to be wondered at that an nworthy class of people are frequently to be ncountered holding supreme authority in homes of well-established reputation which have changed hands so often that the superintendent is hardly known to the doctors who yet must continue to send patients to take their chance under her rule? The wonder is less that thoroughly incapable people are often to be met with in this sphere of life, than that professional swindlers are not more often encountered. It may be surmised that the profits are too precarious and the outlay too large to invite the attention of those who expect to dupe the public to good advantage.

But it becomes an urgent question whether the omission to place houses of this description under public inspection does not now amount to a very serious danger. In the eye of the law, a patient, so he be not suffering from mental disease, is perfectly well able to look after himself, and choose whither he will go to be nursed. If he cannot pay for maintenance during illness the parish offers him accommodation. If he can pay, he is left to the tender mercies of those who earn their living by keeping a species of hotel for the sick. But it is idle to regard him in either case as a free agent. In the first place he does not know, and it is not his business to know, the conditions under which he ought to be nursed. And in the second place, a man who is dangerously ill is, to all intents and purposes, as completely helpless as though he were mad. He is carried hither or thither at the suggestion of some relative, or of some medical man with whom, perhaps, he has but a casual acquaintance. He has every right in the hour of his extremity to the protection extended as a matter of course to the lunatic. The root of the claim for legislation lies then in the defenceless condition of the persons who use nursing homes. The ordinary citizen ought to be safeguarded in respect to places to which he is compelled to resort in illness. Very often it is actual compulsion which drives him there. He may be living in a hotel, in chambers, or under other circumstances precluding the possibility of his being unrsed at "home." He has a right to expect that houses permitted to receive successive cases of a grave nature shall be impeccable in matters of drainage; that the nursing knowledge of his attendants shall extend beyond the art of putting on a becoming uniform; that proper precautions are observed in preparing the room for his reception, and that the household is conducted in accordance with hygienic principles. He is well aware that when inspection has done its utmost there will still be good, better, and best among these establishments. But he craves to have the word "bad" wiped out entirely in this connection, and he is justified in this claim at the hands of the law. For be it remembered the bad nursing home is an infinitely more dangerous spot to be ill in than the most insanitary, noisy and unsuitable private house that could be found in the whole of London. Big operations are performed successfully and good recoveries are made every day in the most pitiable hovels of the poor. Such dangers as exist are readily gauged, and can be provided against one by one. The dirtiest room can be rendered safe for operations when its dirt is conspicuous. But the private home is seldom the abode of disease as well as of dirt. The surroundings may be altogether unsuitable for a sick man, but at least they have not looked down upon a long succession of other diseases. The bed may be insanitary, but it is free from the contagion of other moribund sufferers. It is the septic accidents of life against which a man needs to be guarded in his own home. It is the septic forces of death and disease to which he is exposed in the atmosphere of the bad nursing home. And for this reason legislation ought to make it impossible for this business of deadly danger to be lightly entered upon, unrestrained by licence or inspection, at the whim of any foolish woman who has a few hundred pounds to throw away. Is it too much to ask that the person who aspires to hold the issues of life and death as head of a nursing home should be regarded as holding at least the same measure of public responsibility as the person who dispenses squibs and crackers to little boys, or retails petrol to motorists? In both these cases, with an eye to the public safety, strict regulations are enforced respecting the premises in which such dangerous commodities are to be stored. Will it be contended that cancer, typhoid, and phthisis, to name but a few of the diseases herded under the roof of tne nursing home, are less deadly than gunpowder or petrol? The march of events has been rapid, and the law, which always lags a long way in the rear of civilisation, has hardly yet become aware of the existence of nursing homes. When it once awakes to the gravity of the interests involved, the right divine of the nursing home to nursing wrong will disappear. It wil at last be illegal for unqualified people to endeavour to make money by receiving in unsuitable premises sufferers from acute disease.

